# Cognitive functioning in healthy females with different APOE4 carrier status

**DOI:** 10.1002/alz70855_102114

**Published:** 2025-12-23

**Authors:** Isidora Dzodic, Predrag Aleksić, Maksim Šarčević, Una Lazic, Anja Vrlješ, Ana Lešić, Biljana Salak‐Djokic, Vera Ilic, Gorana Mandic Stojmenovic, Elka Stefanova, Tanja Stojkovic

**Affiliations:** ^1^ Faculty of Medicine, University of Belgrade, Belgrade, Serbia; ^2^ Neurology Clinic, University Clinical Centre of Serbia, Belgrade, Serbia; ^3^ Neurology Clinic, Clinical Center of Serbia, Belgrade, Serbia; ^4^ University Clinical Center of Serbia, Belgrade, Serbia

## Abstract

**Background:**

Over 65% of people with late‐onset Alzheimer's Disease (AD) are women. It is hypothesized that the higher rate of AD in women compared to men may be because women are more negatively affected by some of the known AD risk factors, particularly apolipoprotein E e4 allele (+APOE‐e4).

**Method:**

We recruited 114 healthy female volunteers aged 30‐65 years among employes of the Belgrade University. The exclusion criteria were conditions affecting communicative ability and/or safe engagement in the interventions, as well as presence of any neurological, psychiatric, medical condition or iatrogenic cause known to affect the brain structure and/or function. All participants underwent a detailed assessment including basic demographic data, data on vascular risk factors, mood scales and comprehensive neuropsychological assessment as well as Cognitive Reserve Index Questionnaire (CRIq). Statistical analysis was done using SPSS20.

**Result:**

Average age of all participants was 48,15±8,41 and average years of education was 19,02. In this cohort, which included 27 APOE4 heterozygotes, 2 homozygotes, and 85 non‐carriers. Since there were only 2 homozygotes, we compared heterozygotes to non‐carriers. Heterozygotes had a mean age of 46.27±7.91, and non‐carriers had a mean age of 48,54±8.62. No significant differences were observed when compared neuropsychological measures by domain (i.e., attention, working memory, executive functions, verbal and visual memory, visuospatial and language functions).

**Conclusion:**

We did not find differences in cognitive performance in healthy working‐age females regarding their APOE4 carrier status. More research is needed on this kind off cohorts especially with APOE4 homozygous to better understand the onset of cognitive disturbances connected with APOE polymorphism.

